# A low-profile wideband compressed single-arm spiral antenna array for mid-band 5G beam steering applications

**DOI:** 10.1038/s41598-022-16423-9

**Published:** 2022-08-13

**Authors:** Benjamin J. Falkner, Hengyi Zhou, Wei Zhang, Georgios Metaxas, Amit Mehta, Volker Ziegler, Thomas Multerer, Dariush Mirshekar-Syahkal, Hisamatsu Nakano

**Affiliations:** 1 Swansea University, Swansea, SA1 8EN UK; 2 Airbus Central Research and Technology, 82024 Taufkirchen, Germany; 3 School of Computer Science and Electronic Engineering (CSEE) at University of Essex, Wivenhoe Park, Colchester, CO4 2SQ USA; 4 Department of Electrical and Electronics, Science and Engineering at, Hosei University, Tokyo, 184-8584 Japan

**Keywords:** Engineering, Electrical and electronic engineering

## Abstract

A low profile wideband spiral antenna array is presented for global mid-band 5G beam steering applications. In the global rollout of mid-band 5G, different frequencies have been licensed within each region (e.g. 3.4–3.8 GHz in the EU and 3.7–5 GHz in the USA). Therefore, antenna arrays must be able to cover a bandwidth of 3.3 GHz to 5 GHz to provide true global coverage. Initially, this work presents the design of a wideband compressed spiral antenna that provides an axial beam throughout its operational bandwidth of 3.3 GHz to 5 GHz, enabling beam steering functionality. Then, this antenna has been placed in a 4 × 4 array with a triangular lattice. The proposed spiral antenna array can provide a scanning range of − 40° ≤ θ ≤  + 40° in all azimuth directions with an average back lobe level of less than − 9.5 dB. This development will allow for low-cost integration of 5G systems for global use, such as passenger aircraft, UAVs, drones, and marine and ground vehicles.

## Introduction

The fifth generation (5G) mobile network is at the cusp of becoming the dominant form for wireless communication and internet access. Its advantages over previous generation cellular technology in speed and latency are already enabling new applications of big data and edge-cloud cooperation. However, in order to enable effective adoption of this technology across all industries, it is vital that integration of 5G communication is simple, low cost and comprehensive.

Aerospace and terrestrial multi-point high-throughput links are becoming a particularly challenging, but potentially groundbreaking new use cases for 5G communication. For instance, the internet access currently served to aircraft today is delivered via air-to-ground and satellite hybrid networks such as the European Aviation Network^1^. However, such networks provide limited internet speeds up to 50 Mbps to be shared between all passengers and necessary aircraft communication. By implementing 5G in place of these LTE networks, data rates and latency can be improved by an order of magnitude^2^. Not only does this grant a better experience for passengers for streaming and communication but allows for live data transfer between the aircraft systems and ground services. Such data will enable live remote analysis on the cloud to improve the efficiency, safety, and cost of air travel^3–5^. Furthermore, this data can be sold to third parties such as climate and meteorological research. Unmanned Aerial Vehicles (UAVs) and drones can similarly benefit from wideband 5G coverage as it will enable autonomous drones that can operate cross-border in any region of the world^6^.

Yet, to enable these benefits effectively there are several technical requirements in relation to the antenna performance and design. First, the size and complexity of the design is a key factor of success. A large and complex design can lead to incredibly high fuel costs over the lifetime of the aircraft or an inability to integrate the antenna all together (especially in the cases of UAVs). Hence, attention must be paid to the SWaP-C (Size, Weight, Power and Cost) factor of the design in order ensure it is suitable for productization. Reducing the profile of antenna array must therefore be a priority in order to ease integration and retrofitting while maintaining minimal drag. Secondly, wideband coverage is vitally important for complete global compatibility. While 5G has been predominantly standardized, the frequencies licensed in each region differ (e.g., 3.4–3.8 GHz in the EU and 3.7–5 GHz in the USA)^7^. As such, in order to cover 5G globally with a single system, a bandwidth from 3.3 GHz to 5 GHz^8^ at a minimum is required for mid-band 5G, even though only approximately 0.4 GHz bandwidth is necessary in any one location and its corresponding band. High gain beamforming is also necessary in order to effectively receive and transmit high speed communication over long distances. This further complicates the array design.

Planar wideband antenna arrays are the obvious choice to meet these requirements. However, previous such proposals have encountered a number of challengers that have impeded the development of such a solution. Indeed, existing single element designs such as slotted patches^9–12^ can provide reasonable bandwidth (< 35%), and more complex topologies such as spiral antennas^13–15^, using a traveling wave model, can offer even greater bandwidth coverage (> 50%). However, among these designs the radiation pattern direction can vary drastically across the operational bandwidth. This may be perfectly acceptable for many applications and, indeed, has been successfully exploited for many switch beam steering topologies^16–18^. Alas, for applications such as 5G and LEO satellite communication where array beam steering is required, such variation in unit element beam pattern will heavily restrict the beam steering capabilities of the array. Additionally, wideband beamforming performance will always be impacted by the physical geometry of the array structure as the optimal element distance will change with frequency. Careful optimization of the array geometry is therefore required to ensure consistent gain and steering performance across the bandwidth. To the best of our knowledge, it is for these reasons that there are currently no documented planar spiral antenna arrays capable of providing the wideband beam steering performance presented in this work while maintaining a low profile design.

For instance, some topologies of spiral antenna arrays have been developed that are capable of providing a wide operating bandwidth^19,20^. However, they lack good beam steering due to the large element size and variation of unit element radiation pattern over frequency. A wideband spiral antenna phased array designed by Fang.^19^ demonstrates the challenges mentioned previously and emphasizes that beam steering performance is significantly affected by the variation in unit beam pattern over the operational bandwidth. As mentioned earlier, the array pattern is also impacted by the distance between array elements, and indeed the unit element in the Fang’s work^19^ is larger than λ/2 for most of the operational band. Further, in both Fang’s design^19^ and a wideband array designed by Hovsepian^20^. 4-arm spiral is used that requires a complex feeding structure/network that increases the cost of the system. Another wideband antenna array presented by Hinostroza Sáenz^21^ uses a simpler 2-arm spiral design that is more compact than that of other existing designs^19,20^ and is therefore able to reduce the element spacing to < λ/2. This allows for steering across its bandwidth up to ± 30°. Nevertheless, modern aerospace applications now need beam steering across a range greater than ± 30° not met by Hinostroza Sáenz’s work^21^. Further, to maintain the small antenna diameter and large bandwidth a thick cavity structure (0.35λmax) has been required. Hence, the high drag area of the antenna structure means that even with the reasonable performance demonstrated in Hinostroza Sáenz’s work^21^, the design would be unsuitable for applications such as aerospace and UAVs. Such restrictions, similarly, pose system integration challenges in other new terrestrial high throughput applications. For example, high throughput Low Probability of Interception (LPI), which is presented as an application at the end of the paper.

Dipole designs such as those presented by Hangyu Zhang^22^ and Jian Xu Sun^23^ prove very capable at delivering bandwidths greater than 120% with suitable beamforming capability for 5G applications. However, in order to achieve such performance, these both rely on an antenna array heigh greater than 0.5λ_max_. Again, these are therefor challenging to integrate in many applications.

To solve these challenges, in this paper, a low-profile (< 0.13λ5GHz), compressed spiral antenna array is presented with wideband functionality covering 3.3–5 GHz. This work expands on an antenna design previously developed by the authors of this paper^24^. Here, the optimisation process of this antenna is fully detailed. Further, this element is placed in a 4 × 4 array prototype that is optimised with a triangular topology that provides exceptional scanning capability across the operational bandwidth. This optimised topology is greatly enhanced over the initial antenna array design^24^ and is a key achievement of this work. Here, measurements of this prototype are presented, analyzed, and compared against existing works. With is further optimisation, this work is the first fully documented low profile wideband single-arm spiral antenna array with this level of beam steering performance. Crucial to this work, the spiral antenna element is compressed (spiral diameter < 0.35λmax) such that it provides an axial beam across all operational frequency bands. In summary, this work enables global mid-band 5G with a single, low profile antenna array. The antenna element design and optimization are detailed in Section II. In Section III this proposed spiral antenna element is placed in a 4 × 4 triangular lattice array, enabling beam steering functionality. The array provides elevation steering of − 40° ≤ θ ≤  + 40° at all azimuth angles. The array has then been fabricated and its performance has been measured and analyzed with a bespoke beamforming network. Additional applications and implementation of this work is also provided.

## Antenna element design

### Antenna element structure

**Figure 1:**
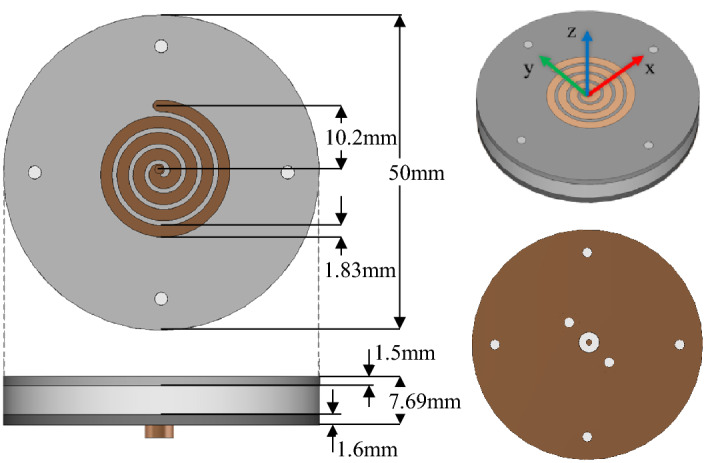
Compressed spiral antenna, its dimensions and top view and side view (left) alongside the perspective and bottom view (right).

A wideband compressed spiral antenna element has been designed and fabricated, providing an axial beam at all target operating frequencies. These properties have been achieved in a low-cost low-profile (< 0.13λ5GHz) design. Figure 1(left) shows the geometry of the design. The antenna is constructed using a stratified medium of three layers. The antenna spiral pattern is milled onto a 1.5 mm thick Rogers RO4350b substrate (εr = 3.48 and δ = 0.0037) at the top of the structure. Added below this is a thicker 4.59 mm layer of Delrin plastic (εr = 2.6 and δ = 0.005). This layer is used as it is low-cost and easy to manufacture into a single thick layer, and its permittivity of εr = 2.6 allows the antenna to be of lower profile compared to an equivalent effective layer of air. Finally, a 1.6 mm layer of FR-4 (εr = 4.8 and δ = 0.025) with a copper plating on its underside is used as a light-weight ground plane. The net height of the spiral antenna is h = 7.69 mm. An SMA connector is used to feed the spiral arm from the bottom of the antenna.

This Archimedean spiral antenna element has a radius of 10.2 mm and a strip width of 1.83 mm. The spiral element has been constructed using the Archimedean spiral equation:

$$\begin{aligned}& r=a\phi \\ & where \,\,\frac{\pi }{2}<\phi <T\times 2\pi\end{aligned}$$

In this design, the spiral amplitude (a) has been set to 0.41 mm/rad and the number of turns (T) has been set to 4.13. This optimized geometry was achieved after several iterations for achieving a stable pattern across a wide frequency band. These optimization iterations are presented in the following section.

### Spiral compression and height optimization

**Figure 2:**
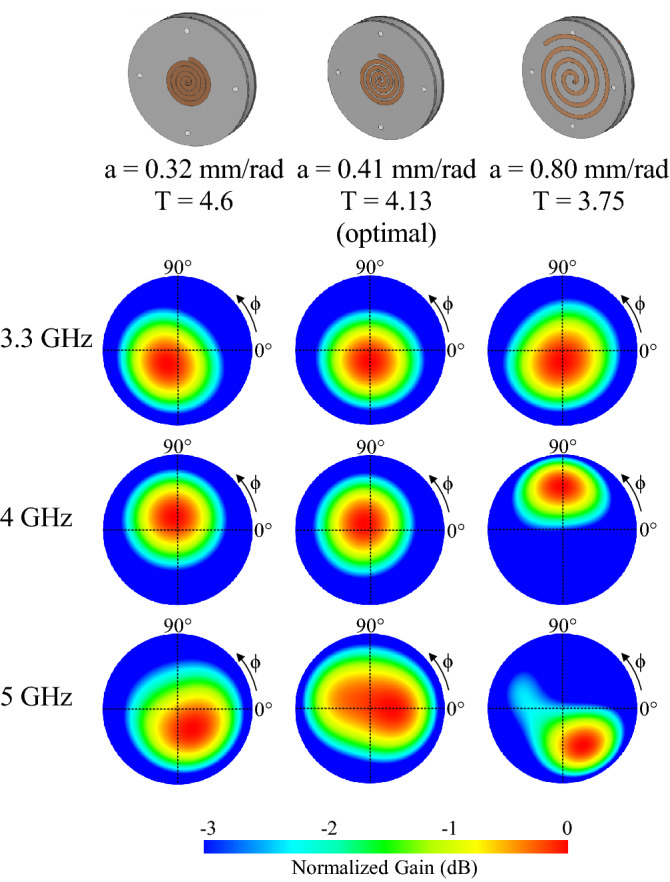
Orthographic radiation patterns of the compressed spiral antenna with a spiral amplitude of a = 0.32 mm/rad, a = 0.41 mm/rad, and a = 0.6 mm/rad across the frequency bandwidth. The optimal design (a = 0.41 mm/rad) shows minimal deviation from the broadside direction.

Compressing the spiral element is a key design feature that has enabled wideband coverage (> 40% bandwidth) whilst maintaining an axial beam. Figure 2 displays an orthographic radiation patterns of an optimized antenna design with a spiral amplitude of a = 0.41 mm/rad, a reduced amplitude of a = 0.32 mm/rad and an uncompressed amplitude of a = 0.65 mm/rad. For all of these variations, a constant ground plane diameter of 50 mm has been used. For a clear comparison, adjustments in the number of turns in the spiral and their orientation have been done to maintain a consistent central frequency of 4 GHz and a broadly close spiral arm end point. Specifically, for the case when a = 0.32 mm/rad, the number of turns is increased to T = 4.6 and inversely, when the amplitude of the spiral is increased to a = 0.65 mm/rad, the number of turns is reduced to T = 3.75.

**Figure 3:**
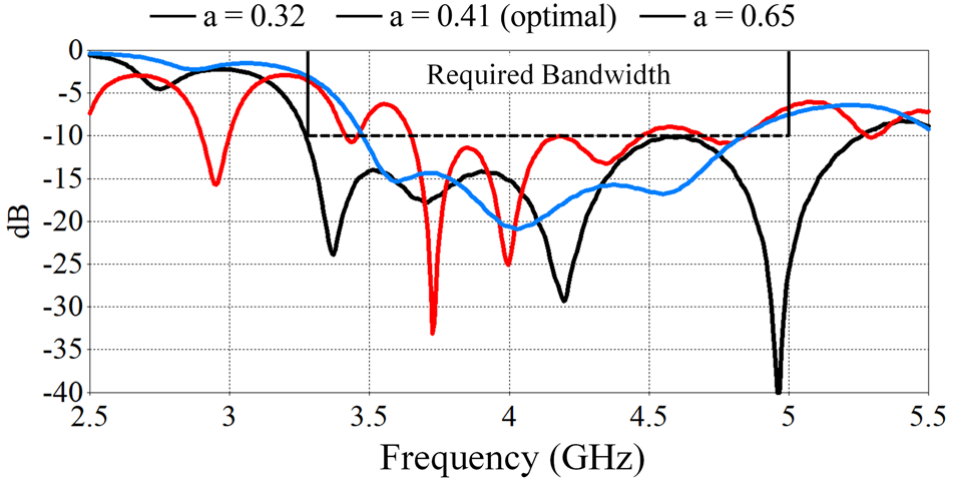
Reflection coefficients of the three-spiral antenna with spiral amplitudes of a = 0.32 mm/rad, a = 0.41 mm/rad and a = 0.65 mm/rad. Only the optimized model with amplitude a = 0.41 mm/rad provides the required bandwidth for the targeted application.

As can be seen in Fig. 2, the selected optimal spiral configuration of a = 0.41 mm/rad produce a nearly constant axial beam across the band of interest. Both pre-optimal a = 0.32 mm/rad and post-optimal (a = 0.65 mm/rad) have pattern deviation from the center. This stable axial beam with frequency will significantly improve the steering performance in an array. Further, it was found that the spiral amplitude and turn variations impacted the resultant reflection coefficient bandwidth. The reflection coefficients of these spiral amplitude variations are presented in Fig. 3. Here it is shown that it is only the optimal design that is able to provide a bandwidth of 3.3–5.25 GHz, which exceeds our targeted applications. The most compressed model (a = 0.32) has a limited bandwidth of 3.5 GHz to 4.8 GHz and the uncompressed model provides a single bandwidth from 3.6 GHz to 4.8 GHz. Aside from the compression of the antenna, the thickness of the substrate is another key factor for the radiation pattern stability and is also investigated next.

**Figure 4:**
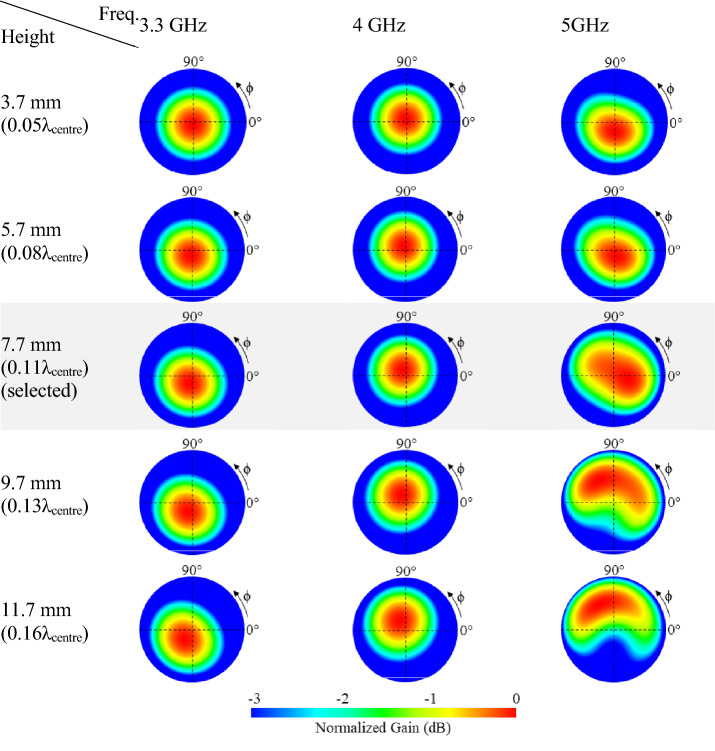
Orthographic radiation patterns of the optimal spiral antenna where the height of the substrate has been varied from 3.7 mm to 11.7 mm with a constant amplitude of a = 0.41 mm/rad and a number of turns of T = 4.13.

**Figure 5:**
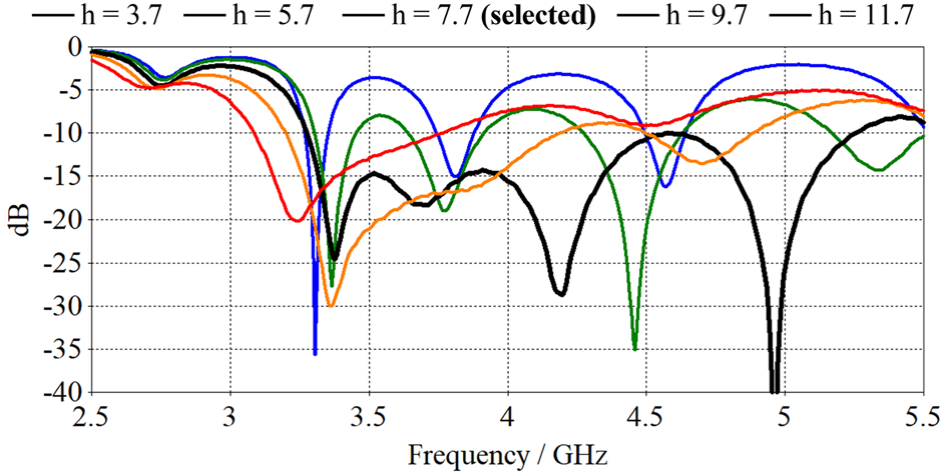
Reflection coefficients of the optimised spiral design with substrate height variation from h = 3.7 mm to h = 11.7 mm.

Figure 4 displays the radiation pattern variation of the optimal spiral as its substrate height is changed from 3.7 mm to 11.7 mm across the target frequency band. The unit pattern remains constant when the substrate is thin (< 0.11λcentre), but it tilts away from the broadside at higher frequencies when the substrate thickness increases beyond 0.11λcentre. However, as with any planar antenna, the substrate thickness plays an important role in the antenna impedance bandwidth. This can be seen in Fig. 5. Indeed, considering Fig. 5, the height 7.7 mm provides the largest bandwidth and a stable axial radiation across the targeted band. Therefore, it was selected for the test design.

### Single-arm spiral simulation and experimental validation

**Figure 6:**
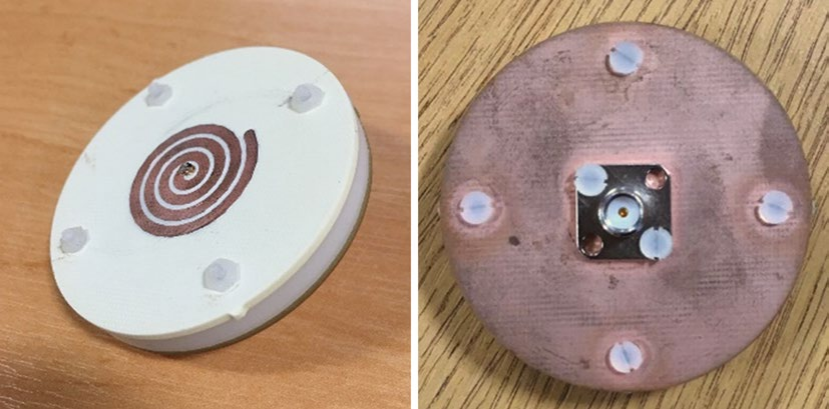
Prototype of compressed spiral antenna; left: front and right: back view.

**Figure 7:**
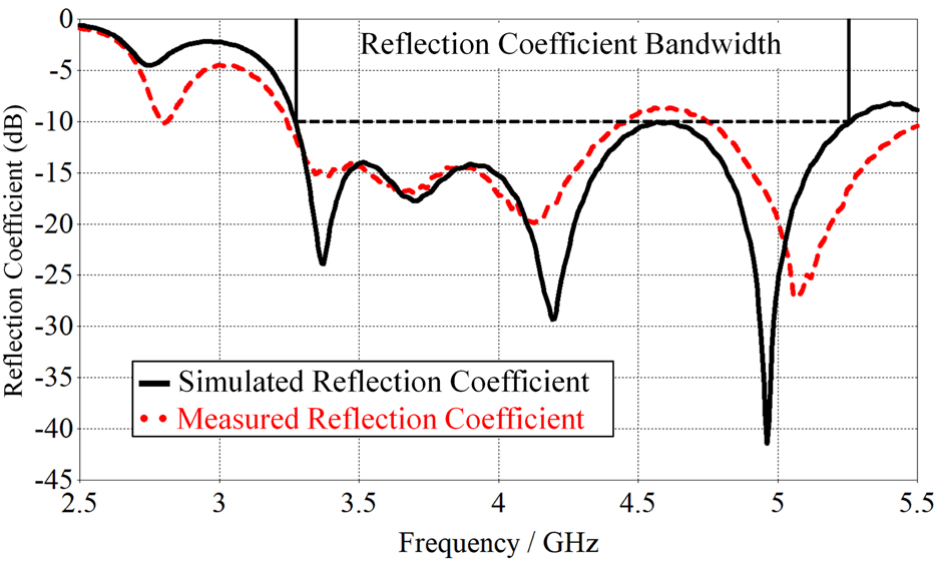
Reflection coefficients for the simulated model and measured prototype of the antenna.

A prototype of the optimal antenna element has been fabricated and measured (Fig. 6). The simulated and measured reflection coefficients of the optimal antenna element are shown in Fig. 7, which are in good agreement. The antenna has a reflection coefficient (S11) bandwidth of 3.3 GHz to 5.25 GHz. The simulated S11 remains below − 10 dB across the entire bandwidth. The measured results match this well, though increase slightly to − 8.7 dB at 4.6 GHz. This can be due to fabrication errors and material tolerances. The antenna prototypes have been measured using a Rhode and Schwartz ZVA 40^25^.

**Figure 8:**
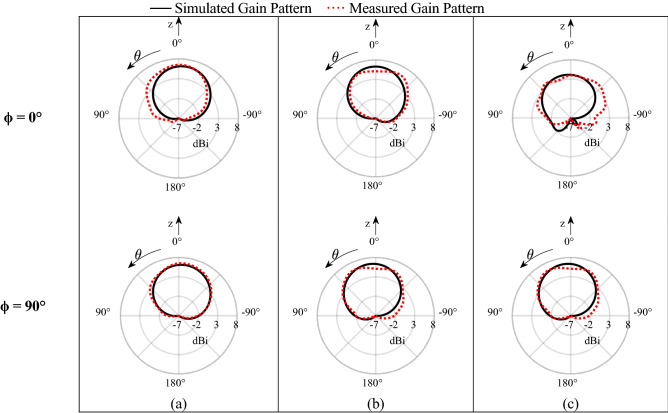
Radiation pattern polar plot cuts of the antenna element at ϕ = 0° and ϕ = 90° at (**a**) 3.3 GHz, (**b**) 4 GHz, (**c**) 5 GHz. The beam remains axial across all required frequencies.

The simulated and measured results which are in good agreement as shown in Fig. 8 verify that the radiation pattern of this antenna element remains axial across the bandwidth 3.3 to 5 GHz. The gain is 6.22 dBi at 3.3 GHz and increases slightly to 6.42 dBi at 4 GHz, and finally drops to 4.31 dBi at 5 GHz. This gain drop at 5 GHz is negated by a natural increase in array gain with frequency due to the longer electrical distance between elements in the array, as shown later in this work. In other words, a reduction in the unit element gain at high frequencies will lead to a more consistent array gain over the bandwidth.

**Figure 9:**
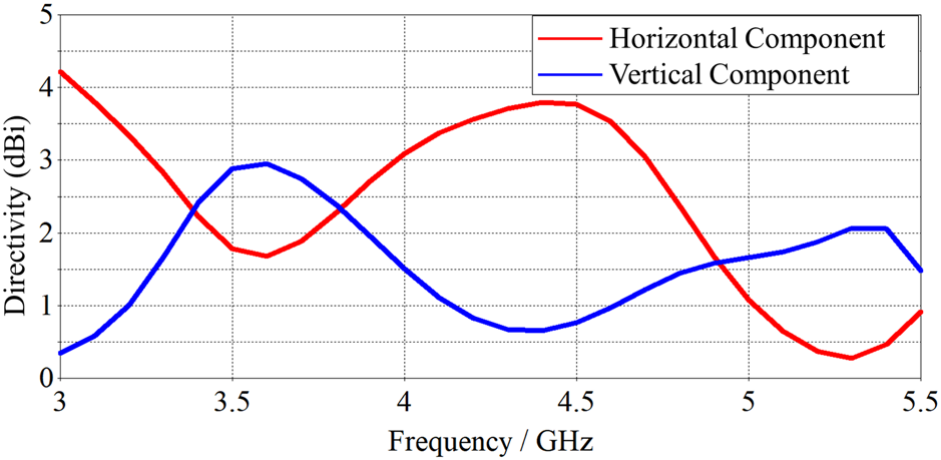
Polarisation components (in the Ludwig 3 coordinate system) of the antenna directivity in the broadside direction.

The polarization of the compressed spiral antenna was found to remain elliptical across its operating bandwidth due to its compact design. Figure 9 displays the horizontal and vertical polarisation components of the antenna over the operating frequency band. From 3.3 GHz to 4 GHz, these component values remain close (Difference < 1.5 dB), as it does from 4.8 GHz to 5 GHz. Between 4 GHz and 4.8 GHz, the horizontal component becomes the primary component and the difference between these reaches 3.2 dB at 4.5 GHz. This polarization profile is suitable for terrestrial communication. While there is some change in the polarisation over the frequency band, this can be resolved by choosing a circularly polarized antenna or a spiral element of the same design at both the base station and the user terminal. The array analysis of the single-arm spiral, its applications and its benefits are presented in the following section.

**Figure 10:**
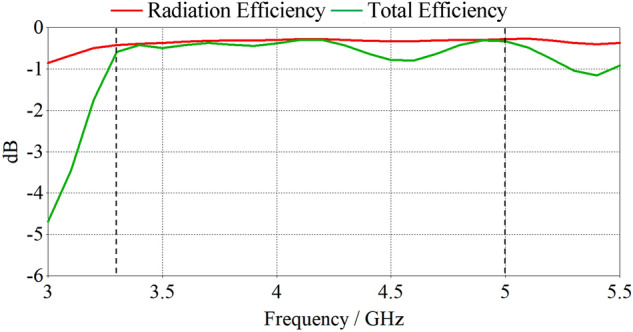
Simulated efficiencies of the optimised spiral design.

Figure 10 displays the simulated radiation efficiency and total efficiency of the optimal spiral antenna element. The radiation efficiency is approximately 0.35 dB across the whole bandwidth. The total efficiency remains above 0.8 dB for the entire operational bandwidth. This is suitable for most modern applications.

## Compressed single-arm spiral antenna array in a triangular lattice design

### 4 × 4 array design

**Figure 11:**
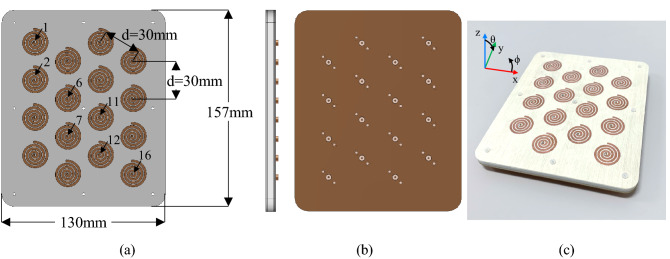
Compressed spiral antennas in a triangular lattice array. (**a**) Design diagram with dimensions and some port locations. (**b**) Side and back view of the antenna array design. (**c**) Fabricated prototype.

The compressed spiral antenna has been placed in a 16-element array configuration in a triangular lattice structure (Fig. 11). This array topology ensures that all neighboring elements have equal distance in all directions, thus reducing the grating lobes when the beam is steered (compared to a square lattice design)^26,27^. Figure 10. shows the array design and the fabricated prototype. The inter-element distance is selected to be 30 mm (varying from 0.33λ3.3 GHz to 0.5λ5GHz). As the compressed spiral element provides an axial beam, this array configuration was found to be able to provide a scanning range of − 40° ≤ θ ≤  + 40° from the broadside (Section C). Indeed, it was found that the average grating lobe levels were better than − 12 dB for + 20° scanning, better than − 11 dB for + 30° scanning and better than − 9.5 dB for + 40°, respectively. These all meet the terrestrial communication needs, which are highlighted in the application section later.

**Figure 12:**
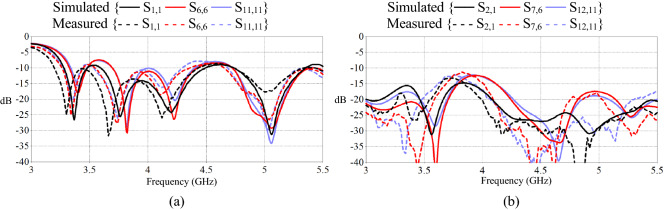
S-parameters of the triangular lattice array. (**a**) Reflection coefficients of ports 1, 6 and 11. (**b**) Isolation between selected ports and neighboring elements 2, 7 and 12.

Figure 12a displays the simulated and measured reflection coefficients of the array when port 1, 6 and 11 are excited. The results are in good agreement and show an adequate reflection coefficient bandwidth ranging over 3.3 GHz to 5 GHz. At a few ports the reflection co-efficient was close to − 7 dB. This was due to mutual coupling, however, for terrestrial applications it does not affect the overall link budget as the minimum acceptable criteria for S11 is − 6 dB (75% transmission).

Figure 12b presents the isolation between three pairs of neighboring elements, i.e.: 1 and 2, 6 and 7, and 11 and 12. It was found that the isolation remains below − 15 dB for most of the operational bandwidth, increasing to − 12.2 dB at 3.9 GHz for S7,6 and S12,11. Again the simulated and measured mutual coupling results were found to be better than − 10 dB cut off criteria. Isolation could be further improved at lower frequencies by the use of a via structure, slotted ground plane^28^ or the addition of a meta surface substrate or superstrate^29,30^.

## Antenna feeding network and beam steering measurement

**Figure 13:**
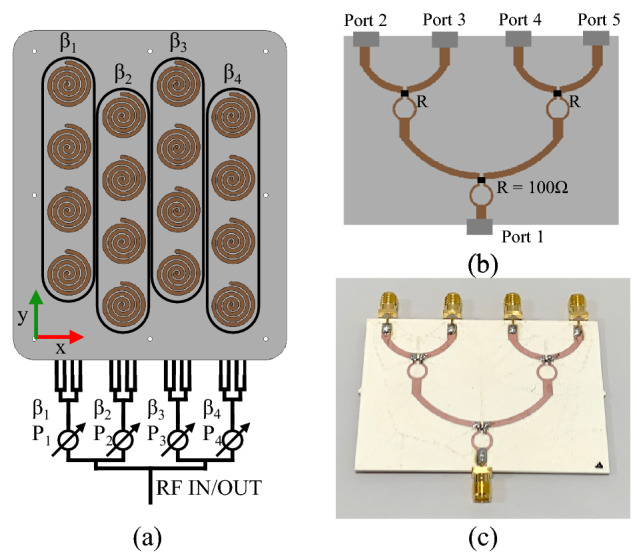
The prototype feeding network, (**a**) feeding network topology, (**b**) 1–4 Wilkinson power divider design, (**c**) 1–4 power divider prototype.

To experimentally examine the beam steering capability of the fabricated array, a feeding network was developed. This feeding network splits the main RF power into four feeding arms. Four phase shifters [P1–P4] are added to these four arms to produce four different phases (β1–β4) in the four columns of the array as shown in Fig. 13a. This allows for elevation beam steering along the x-axis only. In the final operation, individual phase shifters per element using RFICs^31^ can be implemented to enable full azimuth coverage. In this work we used two sets of phase shifters. One set of MACOM MAPS-010164 digital phase shifters were used up to 3.8 GHz and then MACOM MAPS-010165 phase shifters were used at higher frequencies from 3.8 to 5 GHz. Figure 13b shows the bespoke 1–4 Wilkinson’s power divider design, and its fabricated prototype is shown in Fig. 13c.

**Figure 14:**
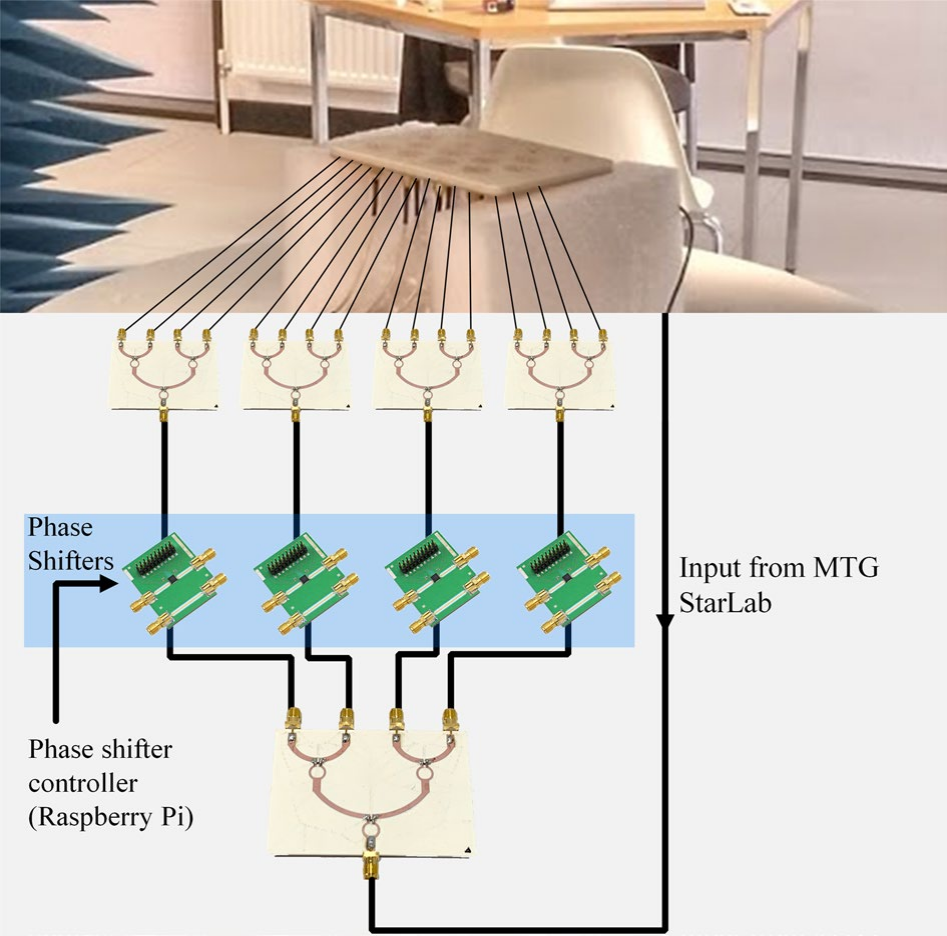
Radiation pattern measurement set-up for the prototype compressed spiral antenna array including the feeding network. Each antenna path is of equal length.

**Figure 15:**
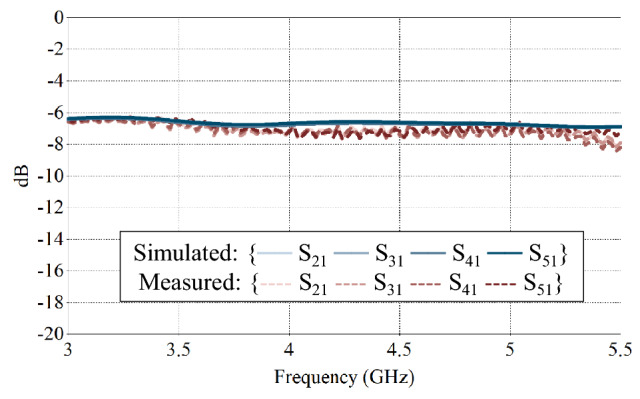
Simulated and measured s-parameters of the 1–4 power divider.

The set-up for the array radiation pattern measurement is shown in Fig. 14. The main RF is split to 16 feeding arms in group of four, each feeding one column. The 1–4 power divider s-parameters are presented in Fig. 15. As expected, the simulated results are close to − 6 dB across the operational bandwidth. The measured results match the simulated results.

## Antenna array beam steering results

**Figure 16:**
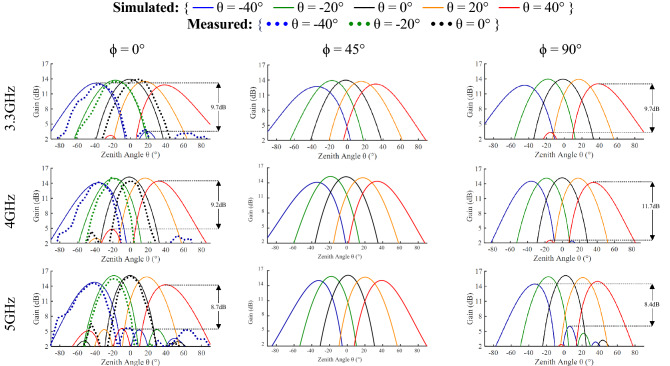
Steering performance as evaluated through simulation and measurement for the 16-element antenna array at ϕ = 0° and ϕ = 90°. Measured data has been normalized in order to remove the impact of feeding network losses.

The simulated beamforming performance of the array in Fig. 16 demonstrates the steering capability of the antenna array for 3.3 GHz, 4 GHz and 5 GHz across the azimuth planes ϕ = 0°, ϕ = 45° and ϕ = 90°. The phase shift values used to achieve the results are shown in Table 1. The simulated gain in the broadside direction of the array varies from 13.9 dB at 3.3 GHz to 16.1 dB at 5 GHz. The array has a radiation efficiency of greater than 88% in all beam steering directions across all operational frequencies.

**Table 1:** Phase shifting values used for beam steering results shown in Fig. 14.

Frequency	3.3 GHz			5 GHz		
${{\varvec{\theta}}}_{{\varvec{m}}{\varvec{a}}{\varvec{x}}}$	0°	20°	40°	0°	20°	40°
$\mathrm{\angle }{\beta }_{0}$	0°	0°	0°	0°	0°	0°
$\mathrm{\angle }{\beta }_{1}$	0°	35°	79°	0°	53°	110°
$\mathrm{\angle }{\beta }_{2}$	0°	70°	158°	0°	106°	220°
$\mathrm{\angle }{\beta }_{3}$	0°	105°	237°	0°	159°	330°

The measured radiation patterns of the array including the bespoke beamforming network are also displayed in Fig. 16 for elevation steering. They are in good agreement with the simulated results while normalized to avoid the impact of losses in the feeding network. An important aspect to note is that the spiral array provides a wide bandwidth steering in which the gain varies by less than 3 dB from θ = 0° to θ = 40°, across all frequencies. The grating lobes vary between 11.7–8.7 dB (average grating lobe level of − 9.5 dB), which is better than the required value for terrestrial communications. There is an anomalous presence of one specific side lobe at θ = − 45° when steering to the boresight direction in the measured results beyond 4 GHz, which is not present in the simulation results. This is likely due to presence of 1–4 power dividers being close to the array, causing some reflection.

Table 2 displays a comparison between this work and other relevant array works. The design presented here provides a number of key advantages simultaneously, an ad. It provides a bandwidth > 40% while allowing for complete azimuth beamforming and steering to ± 40° across the entire antenna bandwidth. This has been achieved in a highly compact structure far smaller than most existing spiral array designs. Furthermore, the spiral presented here is a single arm design and is therefore simpler to integrate with a feeding network.

**Table 2:** A comparison between this work and relevant and state-of-the-art wideband beamforming solutions.

	This work	^32^	^21^	^19^	^20^	^18^	^22^	^23^
Antenna element type	Single arm spiral	Single arm spiral	2-arm spiral	4-arm spiral	4-arm spiral	4-arm curl	Dipole	Coupled Dipole
Element size	0.5λ_max_	0.88λ_max_	0.47λ_max_	1.5λ_max_	0.5λ_max_	0.92λ_max_	0.48λ_max_	0.5λ_max_
Element height	0.12λ_max_	0.27λ_max_	0.35λ_max_	> 0.12λ_max_	0.12λ_max_	0.1λ_max_	0.65λ_max_	0.51λ_max_
Bandwidth (%)	41	20	76	120	69	9.8	120	127
Azimuth steering	Yes	No	Yes	No	Yes	Yes	Yes	Yes
3 dB variation steering angle	± 40°	Not presented	± 30°	Not presented	Not presented	± 70°	± 60°	± 45°
Grating lobe level at 30**°** (dB)	− 9	Not presented	− 8	0	− 6	− 9	− 15	Not presented
Max Isolation (dB)	− 11	Not presented	Not presented	Not presented	− 35	− 21	Not presented	− 47
Max Unit Element Gain	6.42	8.2	3.5*	Not Presented	2*	8	5	5

*Approximation calculated from array gain.

## Applications

**Figure 17:**
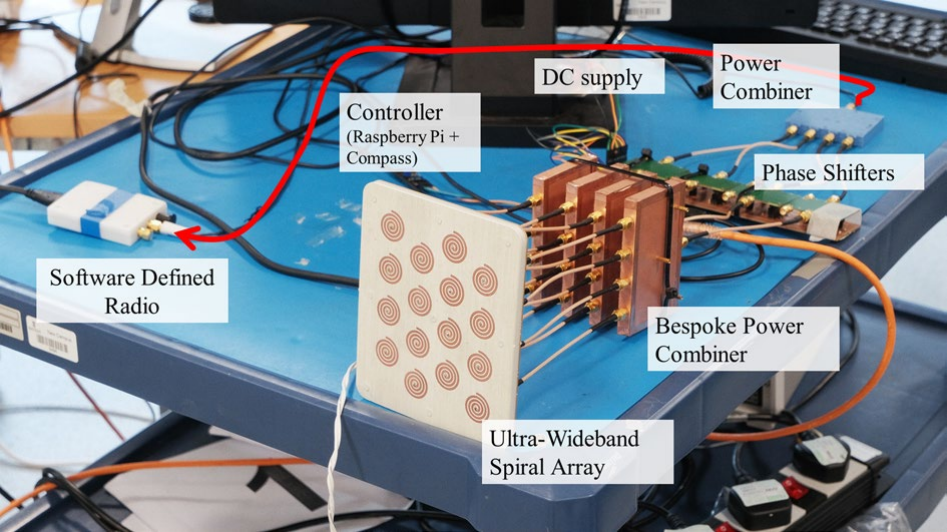
The compressed spiral antenna array test bed for Global 5G and LPI beamforming.

The work presented here has several distinct applications. This spiral array has been designed global 5G coverage for Airbus air-to-ground communications but has also been utilized for high-directivity beam steering for Low Probability of Interception (LPI) communications. The test bed for both these applications is shown in Fig. 17. Here, the wideband, wide beam steering and simple feed structure were the key properties that provided an edge over other conventional systems. The Size, Weight, Power and Cost (SWaPC) of our spiral array were 75% less compared to traditional multiband systems (four times greater bandwidth than a patch array). In addition, the wideband property enabled us to use frequency hopping which is a useful aspect in the list of military communication needs^33^.

## Conclusion

This work presents a wideband phased array consisting of compressed spiral radiating elements arranged in a triangular lattice. The spiral element uses an optimized compressed design to provide 41% bandwidth for coverage of licensed 5G mid-bands from 3.3 GHz to 5 GHz. The unit antenna element generates an axial beam across the bandwidth while having a low profile (0.128λ5GHz) and small size (0.5λ5GHz). When placed in a triangular lattice, a less than 3 dB gain variation in beam steering in range of − 40° ≤ θ ≤  + 40° is achieved with an average sidelobe level of only − 9.5 dB.

The method presented here provides a low profile, low-cost and effective wideband beam steering solution ideal for seamless global 5G applications and for LPI secure communication systems. While this has been developed primarily for terrestrial aviation scenarios, the final work is a light weight and compact design that will also enable usage in small UAVs and ground vehicles for both terrestrial 5G and LEO satellite communication (Supplementary Information).

## Supplementary Information

Supplementary Information.

## Data Availability

The simulated and measured antenna data presented in this paper is freely available from the corresponding author upon request.
